# Periareolar robotic sleeve lobectomy after neoadjuvant osimertinib for epidermal growth factor receptor–mutant lung cancer

**DOI:** 10.1016/j.xjtc.2026.102399

**Published:** 2026-04-20

**Authors:** Ruihao Xie, Lintong Yao, Canjia Cai, Maotao Weng, Luyu Huang, Haiyu Zhou

**Affiliations:** a The First School of Clinical Medicine, Guangdong Medical University, Zhanjiang, Guangdong, China; b Department of Thoracic Surgery, Heyuan People's Hospital, Guangdong Provincial People's Hospital Heyuan Hospital, Heyuan, Guangdong, China; c Department of Thoracic Surgery, Guangdong Provincial People's Hospital (Guangdong Academy of Medical Sciences), Southern Medical University, Guangzhou, Guangdong, China

**Figure:**
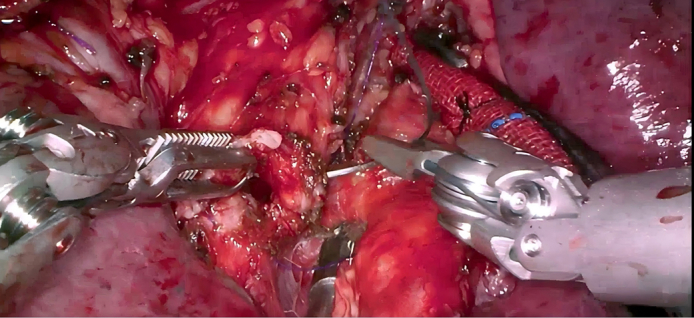
Bronchial sleeve anastomosis on the Edge Robotic system by cosmetic periareolar incision.

**Central Message**

Modified periareolar robotic sleeve lobectomy enables precise reconstruction after neoadjuvant osimertinib, achieving R0 resection without compromising surgical exposure or cosmesis.

Neoadjuvant therapy, which targets micrometastases and R0 resection, is key for the treatment of resectable stage III non–small cell lung cancer. In epidermal growth factor receptor (EGFR)-mutant cases, tyrosine kinase inhibitors (TKIs) like osimertinib induce fibrosis without volumetric reduction, complicating surgery. Robotic-assisted thoracoscopic surgery enhances precision in complex procedures such as sleeve lobectomy. The periareolar reduced-port approach, seldom reported with neoadjuvant TKI, is emphasized here for its feasibility and benefits.^1^^,^^2^

This case report follows the CARE guidelines. Written informed consent was obtained from the patient for publication of this case report and any accompanying images. Institutional review board approval was obtained. The study protocol was approved by academic ethics committees and conducted according to the Declaration of Helsinki and Good Clinical Practice guidelines. The ethics review approval number for this study is no. KY2024-1006-01 (Approved on October 6, 2024).

## Case Presentation

**Figure 1:**
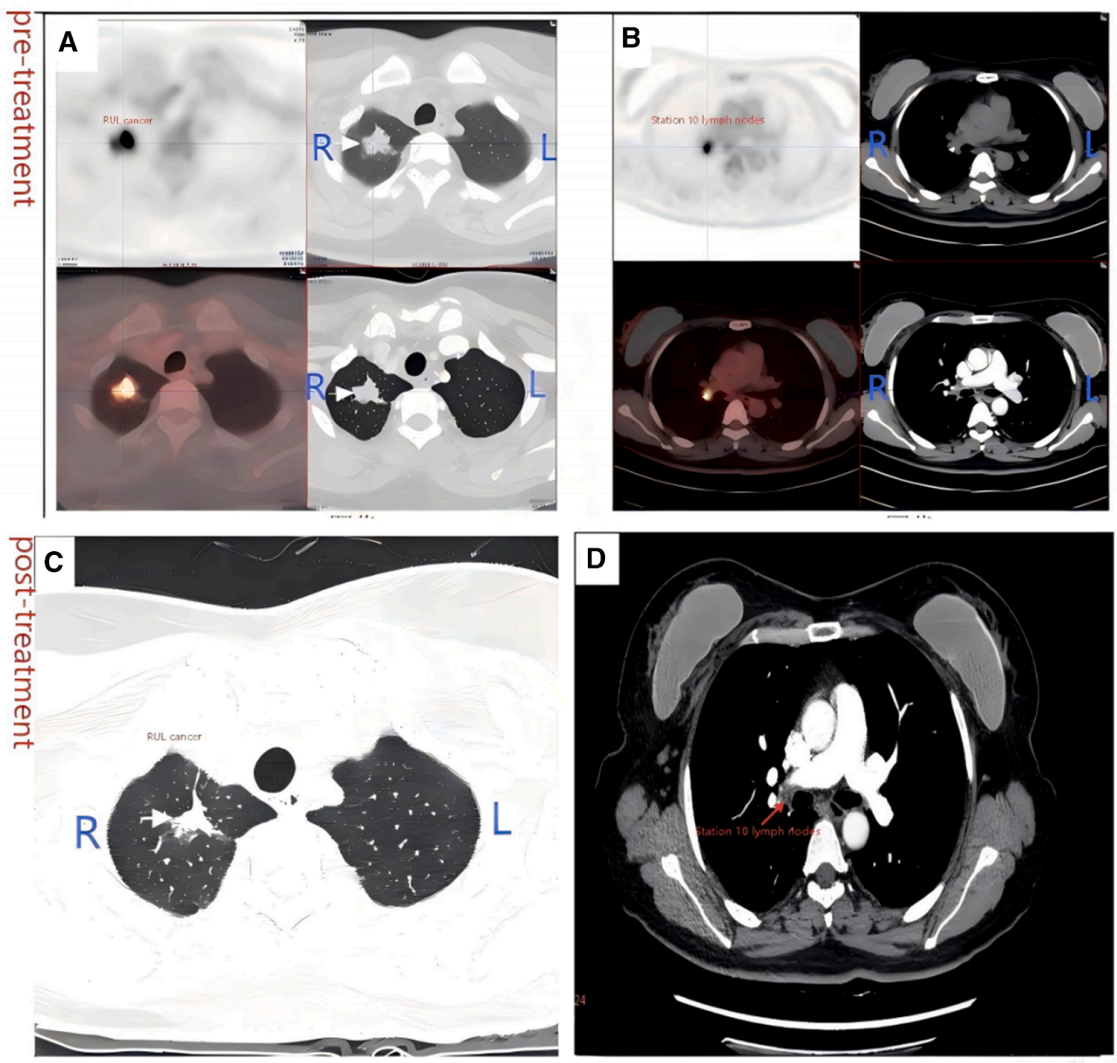
Comparison of imaging before and after treatment. A and B, Pretreatment imaging demonstrates enhancement of the right upper lung nodule and mediastinal lymph nodes. C and D, The radiologic response was evaluated as stable disease after 2 months of osimertinib treatment.

A 47-year-old woman presented with a 2.1- × 1.7-cm nodule in the right upper lobe (RUL). Endobronchial ultrasound–guided transbronchial needle aspiration of stations 4R, 7, and 10R lymph nodes confirmed metastatic invasive adenocarcinoma with micropapillary features and an EGFR exon 19 deletion. The clinical stage was cT1N2M0 (stage IIIA). Baseline PET findings are summarized in Table 1. A multidisciplinary team decision led to neoadjuvant therapy using osimertinib (80 mg, one tablet by mouth once per day) without chemotherapy. After 2 months, computed tomography showed stable disease with no progression (Figure 1). Preoperative metastasis in station 10R lymph nodes raised concern for bronchial involvement, prompting the multidisciplinary team to recommend a sleeve lobectomy for R0 resection. This was confirmed intraoperatively by direct invasion of the RUL bronchus, leading to robotic-assisted RUL sleeve lobectomy.

**Table 1:** PET SUVmax values of primary tumor and involved lymph nodes

Lesion site/lymph node station	Size, cm	Metabolic level SUVmax	Metastasis status
Primary lesion			
Right upper lobe nodule	2.1 × 1.7	8.6	
Regional lymph nodes (suspected metastasis)			
Station 1R (lower neck, supraclavicular and sternal notch)	0.6 × 0.5	3.0	Suspicious
Station 2R (paratracheal)	1.1 × 1.0	6.1	Suspicious
Station 2L (paratracheal)	Not specified	/	
Station 3A (prevascular)	0.9 × 0.9	3.6	Suspicious
Station 3P (retrotracheal)	0.9 × 0.8	4.4	Suspicious
Station 4R (lower paratracheal)	1.4 × 1.1	7.3	Suspicious
Station 4L (lower paratracheal)	Not specified	/	
Station 7 (subcarinal)	1.2 × 0.8	2.8	Suspicious
Station 8R (paraoesophageal)	Not specified	/	
Station 9R (pulmonary ligament)	Not specified	/	
Station 10R (hilar)	1.4 × 1.2	7.7	Suspicious
Internal reference (normal physiological uptake)		SUVmean	
Mediastinal blood pool	–	1.9 (Mean: 1.5)	
Liver	–	2.4 (Mean: 2.0)	
Spleen	–	2.4 (Mean: 2.0)	

*PET*, Positron emission tomography; *SUVmax*, maximum standardized uptake value.

## Surgical Procedure

**Figure 2:**
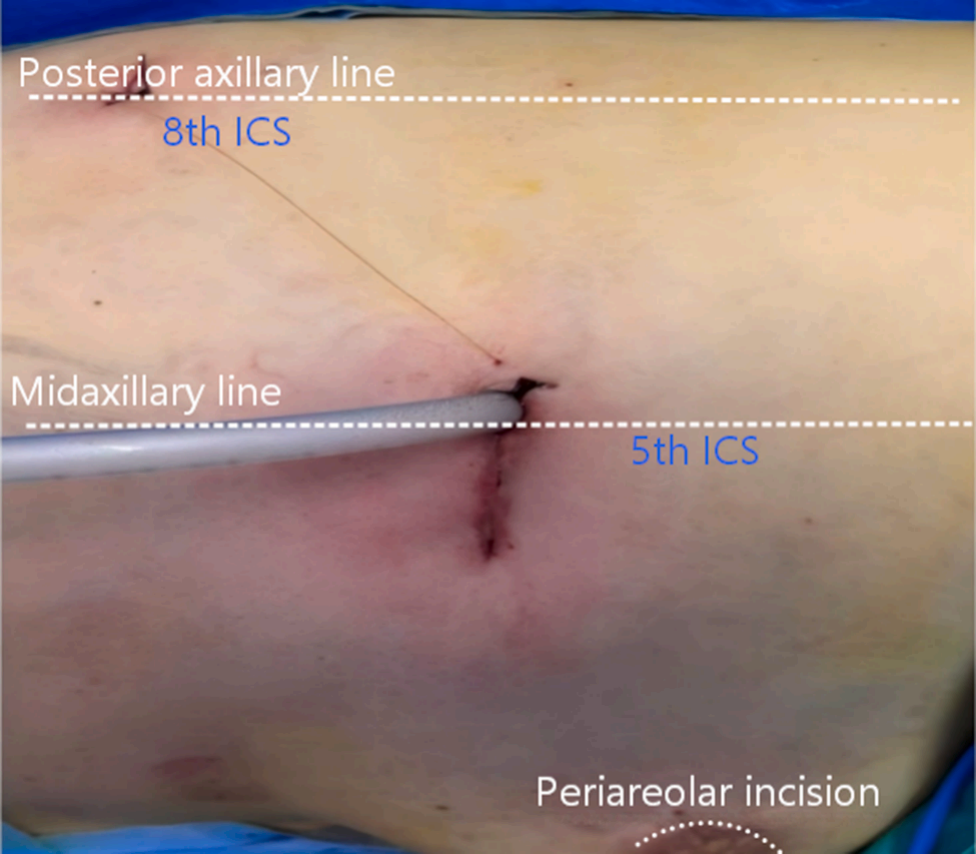
The modified periareolar reduced-port approach. Intraoperative photo shows the trocar configuration and port placement. The 2 operating ports are positioned at the periareolar incision and the eighth intercostal space along the posterior axillary line. A combined camera/assistant port is placed at the fifth intercostal space along the midaxillary line. *ICS*, Intercostal space.

**Figure 3:**
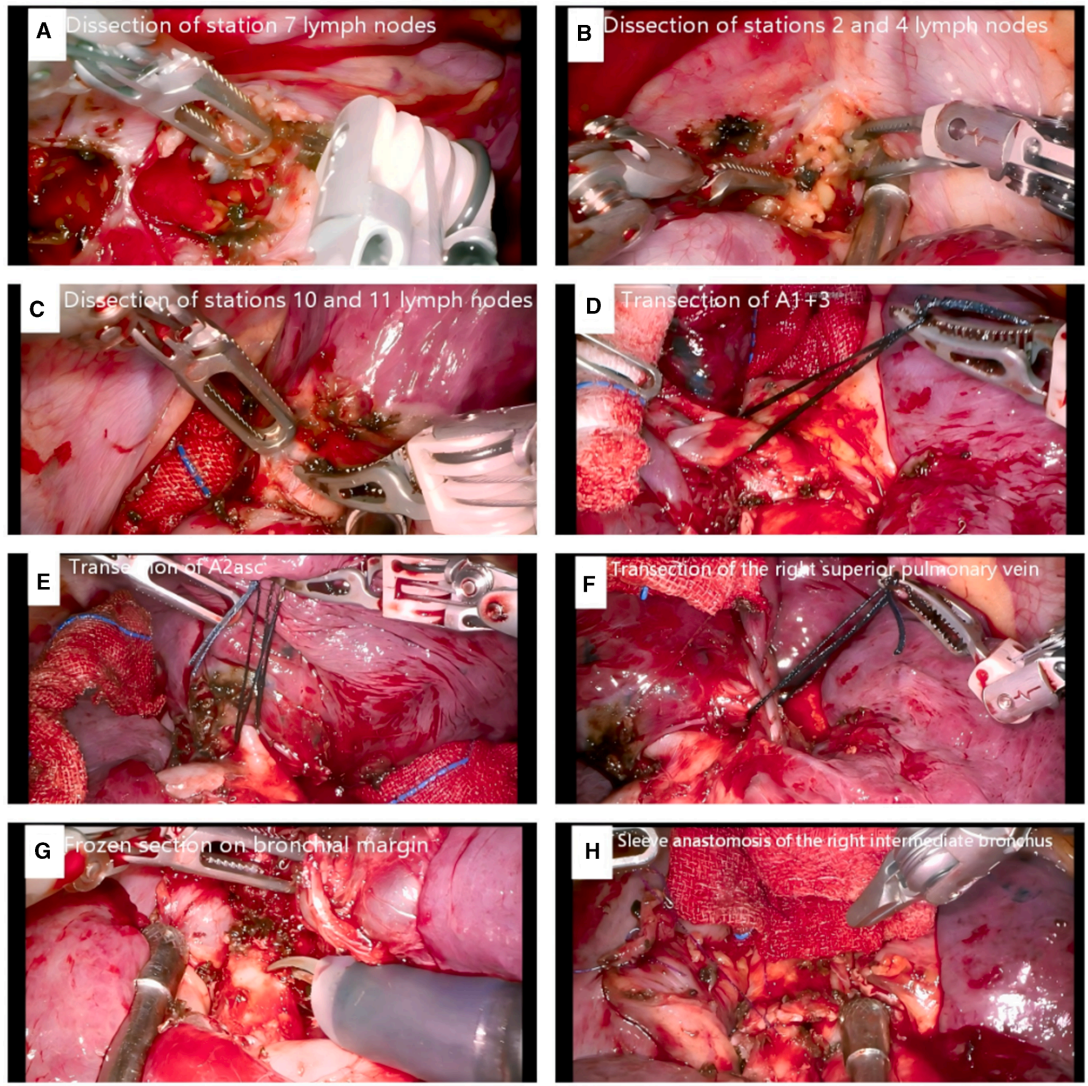
Key procedural steps. A, Dissection of station 7 lymph nodes. B, Dissection of stations 2 and 4 lymph nodes. C, Dissection of stations 10 and 11 lymph nodes. D, Transection of A^1+3^. E, Transection of A^2^_asc_. F, Transection of the right superior pulmonary vein. G, Incision of the right upper lobe bronchus with margin trimming for frozen section analysis. H, Sleeve anastomosis of the right intermediate bronchus.

With the patient under general anesthesia, ports were placed: an 8-mm trocar at the eighth intercostal space (posterior axillary line) for the arm 1; a 3-cm utility incision at the fifth intercostal space (midaxillary line) with a wound protector for the camera and assistant; and a periareolar incision for arm 3. The system was then docked. Lymphadenectomy (stations 2, 4, 7, 8, 9, 10, 11) was performed, followed by bronchial sleeve resection. Anastomosis used barbed sutures; air-leak test confirmed integrity. The azygos vein stump was used to reinforce the anastomosis. Operative time was 124 minutes, and blood loss was 20 mL (Figures 2 and 3, Video 1).

## Postoperative Course and Pathologic Findings

The chest tube was removed on postoperative day 3 per protocol without air leak. Final pathology confirmed ypT2aN2M0 stage with R0 resection. No recurrence occurred during follow-up.

## Discussion

This case demonstrates that robotic sleeve lobectomy via periareolar approach effectively addresses TKI-induced fibrosis.^3^^,^^4^ The technique provides excellent exposure for precise anastomosis while offering cosmetic benefits. Stable disease after osimertinib aligns with the known effects of TKI, where fibrosis may predominant rather than shrinkage. This challenges major pathologic response as the sole success metric, emphasizing R0 resection as paramount.^5^ The periareolar method reduces intercostal nerve injury and enhances recovery.

## Conclusions

Neoadjuvant osimertinib with subsequent robotic sleeve lobectomy via a modified periareolar approach is an effective multimodal strategy for stage IIIA EGFR-mutant non–small cell lung cancer. The robotic system adeptly manages TKI-induced fibrosis to achieve R0 resection, whereas the periareolar technique offers superior cosmetic and functional outcomes without compromising precision, highlighting the value of innovation in minimally invasive surgery.

## Conflict of Interest Statement

The authors reported no conflicts of interest.

The *Journal* policy requires editors and reviewers to disclose conflicts of interest and to decline handling or reviewing manuscripts for which they may have a conflict of interest. The editors and reviewers of this article have no conflicts of interest.

## Supplementary Data

Video 1 Intraoperative video illustrating key procedural steps. Video available at: https://www.jtcvs.org/article/S2666-2507(26)00206-3/fulltext.
